# Analytical Techniques for the Assessment of Drug-Lipid Interactions and the Active Substance Distribution in Liquid Dispersions of Solid Lipid Microparticles (SLM) Produced de novo and Reconstituted from Spray-Dried Powders

**DOI:** 10.3390/pharmaceutics12070664

**Published:** 2020-07-15

**Authors:** Eliza Wolska, Małgorzata Sznitowska, Katarzyna Krzemińska, Maria Ferreira Monteiro

**Affiliations:** 1 Department of Pharmaceutical Technology, Medical University of Gdansk, Hallera 107, 80-416 Gdansk, Poland; malgorzata.sznitowska@gumed.edu.pl (M.S.); katarzyna.krzeminska@gumed.edu.pl (K.K.); 2 The Faculty of Pharmacy, University of Porto, 4050-313 Porto, Portugal; maria.ferreira.monteiro@outlook.com

**Keywords:** cyclosporine, spironolactone, solid lipid microparticles, DSC, AFM, Raman spectroscopy, NMR

## Abstract

Solid lipid microparticles (SLM) can be presented as liquid suspension or spray-dried powder. The main challenge in SLM technology is to precisely determine the location of the active substance (API) in the different compartments of the formulation and its changes during SLM processing. Therefore, the purpose of the research was to assess the distribution of the API and to investigate the nature of the API-lipid interaction when the formulation was subjected to spray drying, with an indication of the most suitable techniques for this purpose. SLM were prepared with two various lipids (Compritol or stearic acid) and two model APIs: cyclosporine (0.1% and 1% *w/w*) and spironolactone (0.1% and 0.5% *w/w*). Physicochemical characterizations of the formulations, before and after spray drying, were performed by differential scanning calorimetry (DSC), atomic force microscopy (AFM), Raman spectroscopy and nuclear magnetic resonance (NMR). The API distribution between the SLM matrix, SLM surface and the aqueous phase was determined, and the release study was performed. It was demonstrated that, in general, the spray drying did not affect the drug release and drug distribution; however, some changes were observed in the SLM with Compritol and when the API concentration was lower. Only in the SLM with stearic acid was a change in the DSC curves noted. Measurements with the AFM technique proved to be a useful method for detecting differences in the surface properties between the placebo and API-loaded SLM, while the Raman spectroscopy did not show such evident differences.

## 1. Introduction

Solid lipid microparticles (SLM) are one of many versatile types of lipid particles with multiple potential applications [1,2,3]. Within the solid lipid particles, three categories can be distinguished: SLN (solid lipid nanoparticles), NLC (nanostructured lipid carriers) and SLM (solid lipid microparticles). SLN, which are the most common subject of research, were introduced in the early 1990s as an alternative drug carrier system to emulsions, liposomes and polymeric microparticles [4]. They were obtained by replacing the oil in an emulsion with a lipid solid at room temperature [5]. The size of SLN are usually 100–400 nm. 

SLM were developed on the basis of SLN, but they are bigger particles, in the micrometers size range (usually, 1–100 µm and even up to 1000 µm) [6,7]. The solid lipid matrix incorporating the API (active pharmaceutical ingredient) ensures a prolonged release of the API, with the mechanism in vivo depending on the degradation of the matrix lipid [7]. SLM can be administered orally, parenterally (e.g., intramuscularly), topically and as eye drops. Due to the larger particle sizes, in comparison to SLN, SLM can provide higher drug loading and more evident sustained drug release effects. With SLM, one can also mask the unpleasant taste of the API or protect it against degradation (i.e., hydrolysis and oxidation).

In the multicompartment systems like SLM dispersions, one can distinguish four domains where the drug may be localized: the matrix of the lipid particle, the surface of the lipid particle (interphase), micelles and water. The API distribution between these phases is a main feature characterizing the formulation [3,8,9,10]. The most common approach is to incorporate the API in the lipid matrix; however, the other locations should also be considered, especially when the effectiveness of drug loading is evaluated. In the case of some SLM or SLN formulations, a significant fraction of the API was found on the surface of the lipid particles [4,9]. This fraction was responsible for the fast drug release phase and the phase of prolonged release related to the fraction of the API incorporated in the lipid matrix [5,8,11]. Unfortunately, even drug substances well-soluble in lipids are often excluded from the lipid matrix during the preparation or storage [12,13]. Most often, these phenomena are associated with the lipid polymorphic transformations and modifications of the interactions between the API and lipids. These transformations can also be induced by processes such as drying. Therefore, a complex assessment is the basis for the development of such formulations.

Special attention should be paid to the characterization of the SLM by using various analytical tools. While the research on various types of lipid dispersions is based on commonly conducted studies of particle size distributions (laser diffraction method or dynamic light scattering), microscopic observations, pH or zeta potential [14,15,16,17,18], the choice of more advanced research techniques is less obvious. The utilization of modern high-resolution analytical techniques might provide an opportunity for an in-depth study of the changes and processes that occur in the SLM formulations during preparation and long-term storage.

The imaging of lipospheres (both SLM and SLN) and the visualization of structural details were made by various techniques of electron microscopy (scanning electron microscopy and transmission electron microscopy) [19,20,21,22]. In particular, SEM is the technique commonly used. Atomic force microscopy (AFM), which is a three-dimensional topographic technique with high atomic resolution, is based on the scanning of a sample surface with a probe and is also used for the imaging of SLN/SLM sizes and morphology, although experimental data from this technique are very limited [22,23,24,25,26]. Moreover, AFM has not been used yet for testing the surface of a single lipid microsphere in order to characterize the drug-lipid interaction.

Differential scanning calorimetry (DSC), X-ray diffraction and high-resolution proton nuclear magnetic resonance (^1^H NMR) spectroscopy are commonly used to investigate the thermodynamic behaviors of lipids in the particles, including polymorphic transition, crystallization temperature and crystallization degree [27]. A less-ordered arrangement of the lipid crystals increases the drug-loading capacity [28], but, on the other hand, the polymorphic lipid transition to the stable form may lead to drug expulsion from the lipospheres during storage [27]. Attempts have also been made to use NMR to deduce the drug distribution and to obtain information on the mobility of the drug molecules within the particles [27,29]. Additionally, structural analyses like Fourier-transform infrared (FTIR) spectroscopy and Raman spectroscopy were utilized to obtain conformational information about the lipid molecules, drug-excipient interactions and chemical compatibility of the components in SLN, SLM and NLC dispersions during storage [21,30,31]. However, to date, no attempt has been made of mapping the surface of SLM using a Raman technique.

In the undertaken research, the main goal was to investigate the interaction of the model APIs with lipids in SLM, while indicating the advantages and limitations of the analytical methods used. As model APIs, cyclosporine A (CsA) and spironolactone (SPIR) were chosen. CsA is a lipophilic cyclic polypeptide (m.w. 1202.6, log P 2.8), and SPIR is a lipophilic steroid drug (m.w. 416.6, log P 2.78), and due to the fact that both substances are practically insoluble in water, they become good candidates for incorporation in SLM. In addition, CsA was selected because, its localization not only in the lipid matrix but, also, on the surface of SLM was demonstrated [8]. In order to demonstrate the potential of different techniques, SLM formulations varying in composition were prepared and tested. The spray drying process was selected as a model process that can change the properties of the lipid carrier and drug-lipid interaction.

Aqueous dispersions of SLM can be converted, by spray drying or lyophilization, into dry reconstitutable powder that can be stored over a long period, without the risk of physicochemical changes characteristic for liquid dispersions, like premature degradation of the particles, chemical degradation of the API or particle aggregation. Spray drying is a method used to produce the microparticles in a solid state, and the resulting powders can be used as such (oral or pulmonary administration) or after reconstitution as a suspension. The latter is used when, for example, a parenteral delivery is considered and the liquid formulation is unstable. Spray drying was also implemented in the preparation of SLN with the aim to obtain powder for inhalations [32,33] or to increase the physical stability during storage [34,35,36]. In a spray-drying process, hot air is used, and, due to the low melting temperature of the lipids, it is a challenge to produce nonagglomerated and spherical ultra-fine SLN or SLM powders with good redispersibility in water [37].

In our study, the impact of spray drying on the quality of SLM, composed of Compritol (glyceryl behenate) or stearic acid, with cyclosporine and spironolactone incorporated in two various concentrations, was tested.

As mentioned above, it is planned that the final result of the conducted research, whose first stage is presented in this article, will not be limited to the preparations of the SLM formulation in a powder form for reconstitution, but, also, a stepwise protocol will be developed that could be utilized for the selection of the most suitable analytical methods when SLM are to be characterized.

## 2. Materials and Methods

### 2.1. Materials

Cyclosporine A (CsA) was obtained from LC Laboratories (Boston, MA, USA) and Compritol 888 ATO (glyceryl behenate) from Gattefossé (Saint-Priest, France). Spironolactone (SPIR), stearic acid and Tween 80 (polysorbate 80) were purchased from Sigma-Aldrich (St. Louis, MO, USA); polyvinylpyrrolidone (PVP) was from BASF (Ludwigshafen, Germany). Methanol and acetonitrile were purchased from Merck (Darmstadt, Germany) and sodium lauryl sulfate (SLS) from POCH (Gliwice, Poland). All other chemicals used were of analytical reagent grade.

### 2.2. Preparation of SLM Formulations

The composition of all tested formulations (placebo or with CsA or SPIR) is illustrated in Table 1. The drug-loaded formulations contained 0.1% or 1.0% (*w/w*) of CsA and 0.1% or 0.5% (*w/w*) of SPIR. All the SLM formulations contained 10% (*w/w*) of a lipid phase and were prepared using a hot emulsification method, as previously reported [8,38]. The mixing process of the lipid phase with the aqueous phase was performed at 80 °C using a high-shear mixer Ultra-Turrax (T25 Janke-Kunkel, IKA Labortechnik, Staufen, Germany). After cooling in an ice bath, the dispersions were stored in a refrigerator.

### 2.3. Spray Drying of SLM Dispersions

Dispersions for spray drying were prepared by mixing equal parts (1:1) of 5% (*w/w*) PVP solution with SLM formulations (Table 1) and stirring them on a magnetic stirrer during spraying. Spray drying of the tested formulations was performed using a Buchi Mini Spray Dryer B-290 (Buchi Labortechnik AG, Flawil, Switzerland). The process parameters were varied according to the lipid type in the formulations. Dispersions with Compritol were dried at an inlet temperature 90 °C, with feed rate 2.4 mL/min. When stearic acid was used, the inlet temperature was 80 °C, with feed spray rate 3 mL/min. The aspirator flow rate was constant at 100%, and the air was used as a drying gas. The resulting spray-dried powders were collected and stored in tightly capped glass jars at room temperature.

### 2.4. Characterization of SLM

The analysis of SLM dispersions was performed as described previously [8,38]. The SLM were observed using the optical microscope (Nikon Eclipse 50i, Nikon Corporation, Tokyo, Japan). The particle size distribution was determined by laser diffraction (Beckman-Coulter LS 13 320, Indianapolis, IN, USA). The pH of the SLM suspension was measured using a validated pH meter (Orion, Boston, MA, USA).

Distribution of CsA or SPIR between the aqueous phase and the lipid core was assessed as described previously [8,38]. The fraction of free drug dissolved in the aqueous phase of the SLM dispersion was determined after ultrafiltration. When SLM formulations were diluted with methanol (lipid matrix remained intact), vortexed and centrifuged, in the supernatant, the API was determined as the sum of the amount located in the aqueous phase and at the surface of the particles. By subtracting from this quantity the quantity found in the aqueous phase (determined after ultrafiltration), the amount of the API localized in the interphase was calculated. The remaining amount was the API fraction incorporated in the lipid matrix.

API in all samples was assayed by high-performance liquid chromatography (HPLC) Prominence LC-2030C 3D (Shimadzu Corporation, Kyoto, Japan). For CsA determination, a LiChrospher 100 RP-18, 250-4 column (Merck KGaA, Darmstadt, Germany) was used and maintained at 80 °C. The mobile phase consisted of acetonitrile/water/t-butyl methyl ether/orthophosphoric acid (520:430:50:1, *v/v*) and was pumped with a flow rate of 2 mL/min. The injected volume was 20 µl, with detection at 210 nm. SPIR was analyzed using a LiChrospher 100 RP-18, 125-4 column (Merck KGaA, Darmstadt, Germany) at 50 °C. The mobile phase consisted of methanol/water/acetonitrile (48:50:2, *v/v*), with a flow rate of 1 mL/min. The injected volume was 10 µl, with detection at 238 nm.

### 2.5. In vitro Release Study

The drug release was investigated by mixing SLM with the acceptor fluid [39,40,41] in the membrane-free system. The appropriate quantity of SLM with CsA or SPIR was suspended in 5 mL of 0.5% SLS and incubated under agitation at 37 °C. At the predetermined time intervals, samples were taken, filtered through a 0.2-µm filter (cellulose acetate filters, Sartorius, Goettingen, Germany) and diluted with methanol. The concentration of CsA or SPIR dissolved in the acceptor fluid was determined by HPLC method.

### 2.6. Thermogravimetric Analysis

Thermogravimetric Analysis (TGA) was carried out using Mettler Toledo TGA2 (Mettler Toledo, Greifensee, Switzerland) instrument. The TG curves were recorded at the temperature range of 25–200 °C under an atmosphere of nitrogen (50 mL/min) and at a heating rate 10 °C/min. The obtained curves were analyzed with STAR^e^ Software 14.00 (Mettler Toledo, Greifensee, Switzerland).

### 2.7. Differential Scanning Calorimetry

Differential scanning calorimetry (DSC) measurements were carried out with a DSC1 Mettler Toledo instrument (Mettler Toledo, Greifensee, Switzerland). The samples were placed into aluminum pans under a nitrogen flux (50 mL/min) and heated from 25 °C to 200 °C at a scanning rate of 10 °C/min. For comparison, the same procedure was followed for the raw materials (lipids, API and polymer). Each sample was analyzed in duplicate. DSC curves were analyzed with STAR^e^ Software 14.00.

### 2.8. Raman Spectroscopy Measurements

Raman spectra acquisition and Raman mapping were generated for all tested formulations, both placebo and with the API, using a confocal Raman microscope WITec Alpha 300 (WITec, Ulm, Germany). For these experiments, a laser beam at 785 nm with a power of 130 mW at the source was employed (in the initial stage of research, a laser power of 100 mW was used). The spectra of the individual components, as well as the liquid (marked in the figures with the abbreviation FL and corresponding to SLM dispersions) and the solid (marked in the figures with the abbreviation FS and corresponding to spray-dried powders) formulations, were acquired. All the spectra were obtained in the spectral range of 290–1740 cm^−1^, with approximately 3 cm^−1^ resolution. The measured area varied from 170 µm × 170 µm to 1 mm × 1 mm when the clusters of individual microspheres were analyzed.

### 2.9. Proton Nuclear Magnetic Resonance (^1^H NMR) Measurements

Proton Nuclear Magnetic Resonance (^1^H NMR) spectra of samples prepared in deuterated water were obtained using a NMR700 MHz spectrometer (Bruker, Bremen, Germany) operating at 300 K.

Deuterium oxide was added to the samples prior to the measurements. The samples were prepared by diluting the dispersion (FL formulations) with D_2_O in a 1:1 ratio or by dispersing SLM in water (FS formulations) and then diluting with D_2_O in the same ratio. Prior to the analysis, there were prepared samples of each dispersion in aliquots approximately 0.5 mL placed in NMR tubes.

In addition, two liquid formulations (F6 and F8) containing PVP were also tested (PVP was added in the same concentration as in the dried formulations; see Table 1).

### 2.10. Atomic Force Microscopy (AFM) Measurements

AFM studies were done in a Nanosurf Flex-Axiom system (Nanosurf AG, Liestal, Switzerland), and data analysis was performed with AtomicJ software.

For AFM investigations, the liquid formulations of SLM were placed on a glass surface, and the sample was then stored in a desiccator at room temperature in order to remove the water. Spray-dried SLM were fixed onto a metal disk using double-sided tape.

AFM analysis was performed to measure the interaction forces between the tip and the sample surface. The experiments were done in an ambient temperature operating in a contact mode. Pyramidal cantilever with spherical gold probe (sQUBE, Bickenbach, Germany) with a diameter of 4 µm and spring constant of 3.8 N/m was used. For the force measurements, the cantilever was moving only in the z-direction. The force measurements were made by collecting the force distance curves, which are the plot of cantilever deflection as a function of the tip-sample separation [42].

## 3. Results and Discussion

### 3.1. Characterization of the SLM Formulations

SLM dispersions were prepared with two types of the lipids (Table 1): Compritol (mixture of mono-, di- and tribehenate of glycerol) and stearic acid (long-chain saturated fatty acid). Both lipids are classified as “generally recognized as safe” (GRAS) by the Food and Drug Administration (FDA) [43], and they have similar melting points higher than body temperature (69–74 °C).

The research was designed to enable identification of the physicochemical interactions between the lipids and the incorporated API during the spray drying of SLM and the characterization of changes in the API distribution as a result of these interactions. Bearing in mind this goal, three factors differentiating the formulations were selected: lipid (Compritol or stearic acid), the model API (cyclosporine or spironolactone) and its concentration (0.1% or 1% for CsA and 0.1% or 0.5% for SPIR).

**Figure 1:**
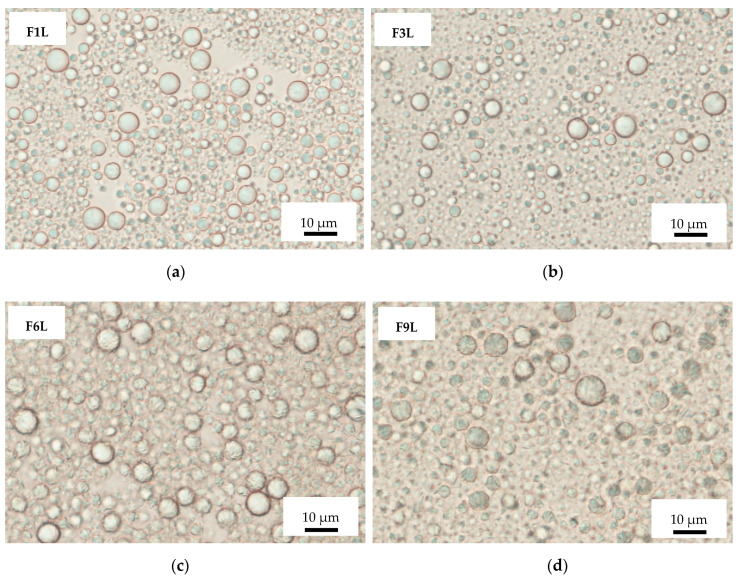
Optical microscopic picture of solid lipid microparticle (SLM) formulations: placebo F1L (**a**) and F6L (**c**), with cyclosporine F3L (**b**) and spironolactone F9L (**d**).

In the obtained SLM dispersions examined under a microscope, the particles appeared round-shaped, and the majority were of sizes in the range from 1 µm to 8 µm, with none exceeding 15 µm (Figure 1 and Table 2).

**Figure 2:**
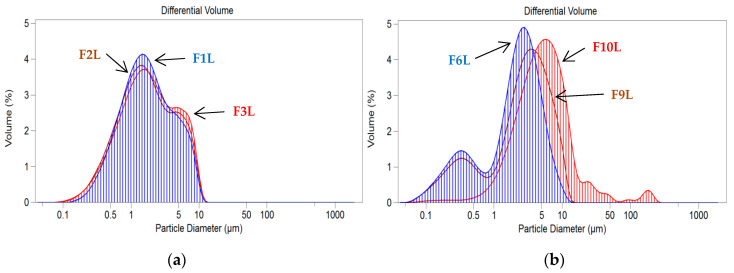
Particle size distributions in SLM formulations with Compritol (**a**) and stearic acid (**b**): placebo (F1L and F6L), with cyclosporine (CsA) (F2L and F3L) and with spironolactone (SPIR) (F9L and F10L).

The presence of the 0.1% API did not affect SLM size, while the addition of the API at a higher concentration (0.5% SPIR and 1.0% CsA) resulted in a slight increase in the sizes of the particles (Table 2). Examples of particle size distributions of the placebo SLM and formulations with the model API are presented in Figure 2. As summarized in Table 2, the SLM dispersions were slightly acidic (5.2–5.9) after preparation, which may be due to the presence of free fatty acids [44].

In the distribution study, a large fraction of CsA (from 71% to 76%) was found on the surface of the lipid particles in the interphase. This fraction was easily dissolved after the shaking of SLM with methanol (Section 2.4.) [8]. In all the tested formulations with CsA, less than 1.5% of the total drug content was localized in the aqueous phase. The rest of CsA (22–29%) was identified as incorporated in the lipid core. These observations were independent of the lipid type (Compritol or stearic acid) and the API concentration (0.1% or 1.0%). The only exception was the F2L formulation, in which slightly less (around 52%) of CsA was detected in the interphase, and a larger fraction (48%) was located in the lipid matrix. Moreover, only in this formulation were significant changes in the API distribution observed after the spray drying, while, in other formulations, the CsA distribution remained unchanged (Table 2). This indicates that the events occurring during spray drying (e.g., dehydration, collision of particles and melting) do not adversely affect the interaction of CsA with lipids. Although, in the F2 formulation, there was a decrease in the CsA fraction incorporated in the lipid matrix, with a simultaneous increase in the amount located in the interphase, no secretion of CsA in the aqueous phase, nor crystallization, was observed. Therefore, it can be stated that there is no significant effect of the spray drying on the distribution of CsA in SLM.

The results of the SPIR distribution study in SLM (Table 2) are less obvious, especially in formulations with a higher concentration of SPIR (0.5%). This can be related to the poor solubility of SPIR in the tested lipids, with the resulting limited incorporation of SPIR in the SLM matrix. Although both tested API are practically insoluble in water and have a similar value of log P coefficients (CsA—2.8 and SPIR—2.78 [45]), only CsA is freely soluble in oils. In the SLM dispersions with SPIR at the concentration 0.5% (F5 and F10 formulations), the crystals of the API were observed. This fact can significantly disturb the results obtained in the distribution test, mainly due to the heterogeneity of the formulation. Focusing on the analysis of formulations with a lower concentration of SPIR (0.1%), the difference in the amount of API in the interphase depending on the lipid type is clearly visible. When SLM were prepared with stearic acid (F9), 57% of the total SPIR content was incorporated in the lipid, and approximately 40% was localized in the interphase. When Compritol was used (F4), only about 20% of the SPIR was in the lipid matrix, and most of the dose (77%) was on the surface of the lipid particles (Table 2). After spray drying, the separation of SPIR between the interphase and lipid core slightly changed (by several percent). At the same time, even about 4% of the total SPIR content was found in the aqueous phase, both before spray drying and in the reconstituted dispersions. In summary, in SLM with SPIR, there is only a slight impact of the drying process on the API distribution visible, depending on the lipid type.

### 3.2. In Vitro Release Study

SLM are multicompartment drug carriers that allow for the prolonged release of API with the aim to increase its effectiveness [8,38]. As was previously reported [38], SLM dispersion with 2% of CsA was characterized by a two-stage release profile: rapid and prolonged, which resulted from the specific distribution of CsA; it was localized mainly on the lipid surface, not being fully incorporated into the lipid core [8,38].

**Figure 3:**
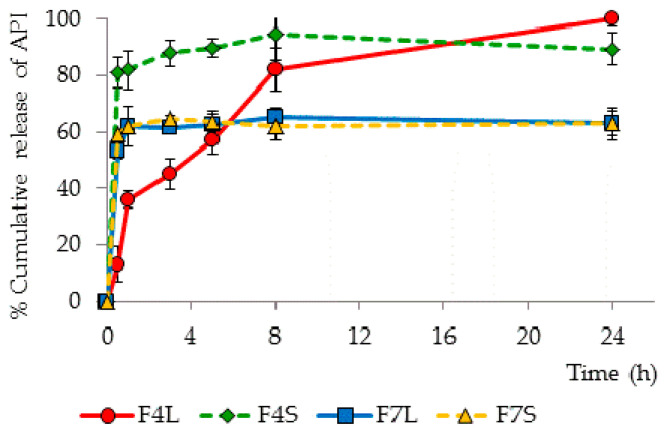
Release profiles of CsA (F7L and F7S) and SPIR (F4L and F4S) from selected SLM formulations before (FL—liquid formulations) and after (FS—spray-dried formulations) spray drying. API: active pharmaceutical ingredient.

The release rate of CsA and SPIR from the tested SLM formulations before and after spray drying is presented in Table 2. Selected release profiles of CsA and SPIR from liquid and spray-dried SLM formulations are presented in Figure 3. The results demonstrate that spray drying of SLM dispersions is possible without affecting the release rate of the incorporated API; however, the type of lipid was the basic factor determining the lack of changes in the obtained release profiles of CsA. Formulations with stearic acid (F7 and F8), regardless of the API concentration (0.1% or 1.0%), released similar amounts of CsA from both the SLM dispersion and the reconstituted powder (Figure 3). However, when Compritol formed the lipid matrix, spray-dried CsA powders released about 20% more API at a particular time point (in F2S, even 40% more CsA was released after 30 min). Similar relationships were observed in formulations with 0.1% SPIR (F4, F9), while dispersions with 0.5% of SPIR were not considered for the aforementioned incomplete incorporation of the API.

It is essential to compare the amount of API determined in the interphase with the release profiles, because the fraction localized on the surface of particles should be released faster. It was confirmed that, in all formulations, the burst effect (at least 50% of the dose released during 30 min) can be related to the large fraction of the API determined in the interphase (40–75%). However, a better correlation of these two parameters was observed in the spray-dried formulations. The most significant change occurred in SLM F2 and F4, containing Compritol and low concentrations of the API (Figure 3). These were the only formulations that released CsA and SPIR without a large burst effect, but after spray drying, the fast release correlated with the large fraction of the API present in the interphase.

One can conclude that, although spray drying of the SLM is possible without a significant effect on the release profile, this is not a rule and depends primarily on the type and properties of the lipid used. No change in the release profile was observed in the SLM with stearic acid, while, in the formulations with Compritol, the spray-drying process led to a faster release of the API.

It is also noteworthy that the effect of spray drying on the Compritol formulations was clearly visible in the release studies, regardless of the CsA concentration, while the differences in the distribution tests were only noticeable at the lower CsA concentration (0.1%).

### 3.3. Differential Scanning Calorimetry

DSC is a thermal analysis technique used to measure enthalpy changes in a sample as a function of temperature or time, very often supported by thermogravimetry (TG) [46]. Such measurements can provide qualitative and quantitative information about physical and chemical changes in the tested material involving endothermic and exothermic processes.

**Figure 4:**
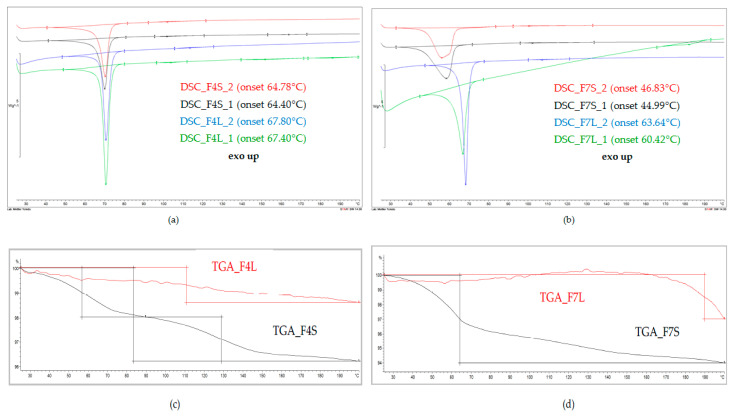
Thermal analysis of the API-loaded SLM prepared “de novo” (FL) and after spray drying (FS): (**a**) and (**b**) are differential scanning calorimetry (DSC) and (**c**) and (**d**) are thermogravimetry.

In our studies, DSC and TG analyses were performed to detect interactions between the API and lipid carriers, particularly as the result of the spray-drying process. Selected DSC and TG curves discussed in this section and representatives of the tested formulations are presented in Figure 4.

The DSC curve of the bulk Compritol presented a single endothermic peak at 73.36 °C (onset at 70.06 °C), which resulted from the melting of the lipid [47]. The tested placebo SLM formulations exhibited similar single melting peak, with no other peaks on the thermograms. In both F1 placebo formulations (F1L: prepared “de novo” and F1S: after spray drying), this peak was found at the same position, being only slightly shifted (less than 3 °C) in comparison to the DSC peak of the pure lipid. This shift may probably have arisen as a result of the change of the lipid from a more stable β polymorphic form into a less stable β’ or α, and the DSC thermograms have proven that the transformation occurred already at the preparation stage of the SLM and was not caused by spray drying [19,43,47]. When CsA or SPIR was incorporated into the Compritol matrix of SLM, no significant changes in DSC curves were observed, regardless of the type of the API and its concentration (F2–F5 formulations “de novo”). The characteristic endothermic peak of the α/β’ lipid form at 70–71 °C was clearly seen in DSC curves of the spray-dried F2-F5 formulations, and no further changes were observed (Figure 1).

The DSC curve of the bulk stearic acid presents, similar to Compritol, a single endothermic peak at 70.71 °C (onset at 67.68 °C) [43]. In comparison to the bulk lipid, significant differences were observed in the thermograms of the F6 placebo formulation prepared “de novo”: a broad endothermic peak was observed, which shifted to a much lower temperature, i.e., to 55.47 °C (onset at 47.71 °C) or 55.14 °C (onset at 47.18 °C) (first and second repetition). This shows that the stearic acid, when compared to Compritol, is definitely more unstable already at the stage of SLM preparation by the hot emulsification method. Polymorphic transformations are often shown by long-chain saturated lipids, such as stearic acid [43]. Interestingly, this change did not occur in some of the drug-loaded formulations. DSC curves of SLM with 0.1% of CsA (F7L) and with SPIR (F9L and F10L) still presented the peak characteristic for the bulk stearic acid, only slightly shifted (less than 3 °C) towards a lower temperature. However, in all spray-dried formulations, DSC curves did not show a peak characteristic for the bulk stearic acid. Instead, a broad melting peak, shifted by more than 10 °C towards a lower temperature, was observed. This means that, even if, in the SLM, prepared “de novo” stearic acid retained its original polymorphic form, the spray-drying process each time affected the crystalline structure of this lipid matrix (Figure 4).

None of the DSC curves exhibited the characteristic peaks of the API (Figure 4). At the same time, the TG curves excluded the risk of API degradation, confirming the thermal stability of CsA and SPIR heated up to 200 °C (loss of weight—Δm not more than 1%). Although the lack of the peak of CsA on the thermograms of SLM formulations may result from the low concentration of the API, this can also result from the interaction of the API with the lipid matrix. The API can be dissolved in the lipid rather than be incorporated as a solid phase in the imperfections of the lipid crystalline structure [44,48]. Additionally, CsA localized in the interphase of SLM can be solubilized. This assumption could justify the high physical stability of the SLM dispersion with CsA without any drug expulsion or crystallization during long-term storage [8].

Thus, the interpretation of the obtained DSC thermograms allowed for the detection of the changes in the lipid structure occurring during spray drying, but the effect on the API-lipid interaction was not detected.

### 3.4. Raman Spectroscopy

Raman spectroscopy detects the vibrations of molecules after excitation by an intensive laser beam [30]. This technique has already been used as a tool to identify and localize specific components in various liquid and solid dosage forms [31,49,50,51]. However, the use of Raman spectroscopy to characterize the colloidal and microparticulate lipid systems is rare [52,53,54].

Based on the results of distribution and release studies (Table 2), it was concluded that CsA and SPIR are mainly surface-localized, and it can be expected that Raman spectroscopy is a suitable technique to confirm this thesis.

**Figure 5:**
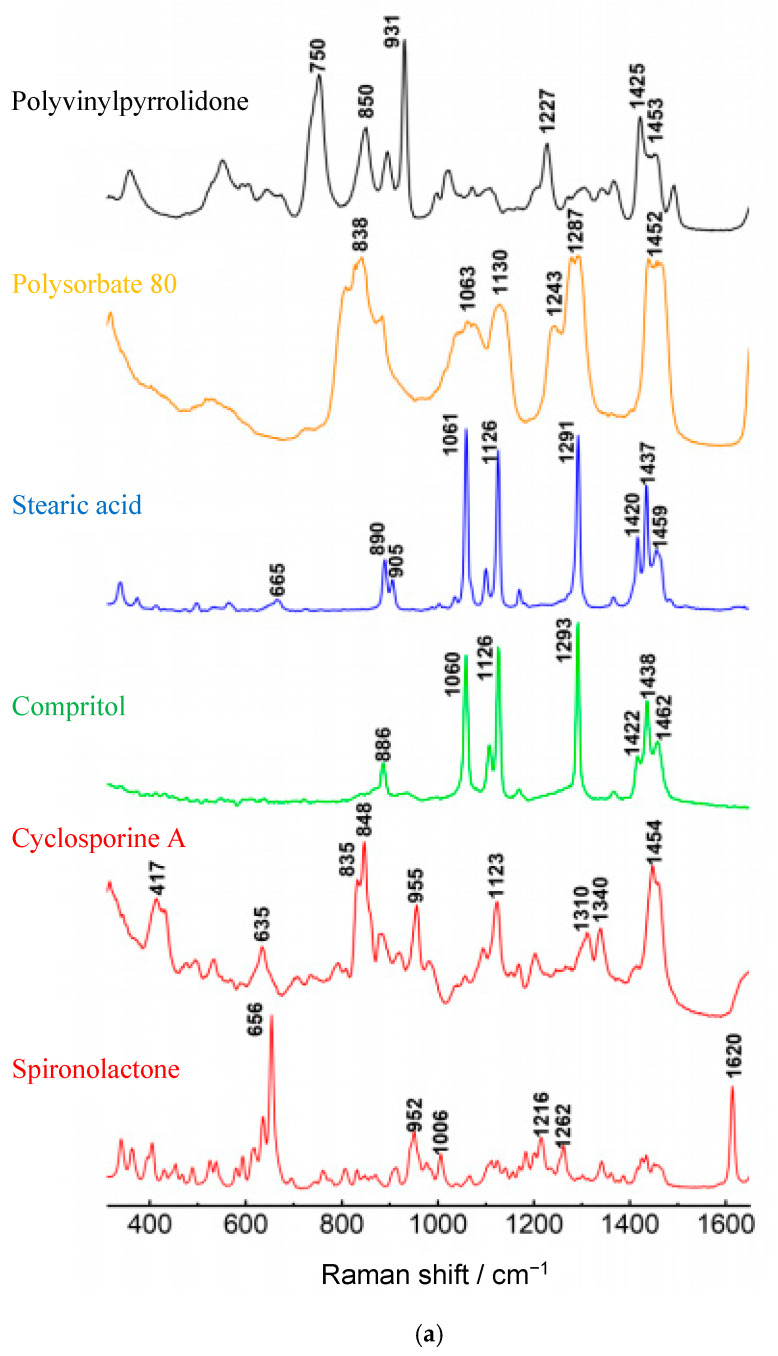

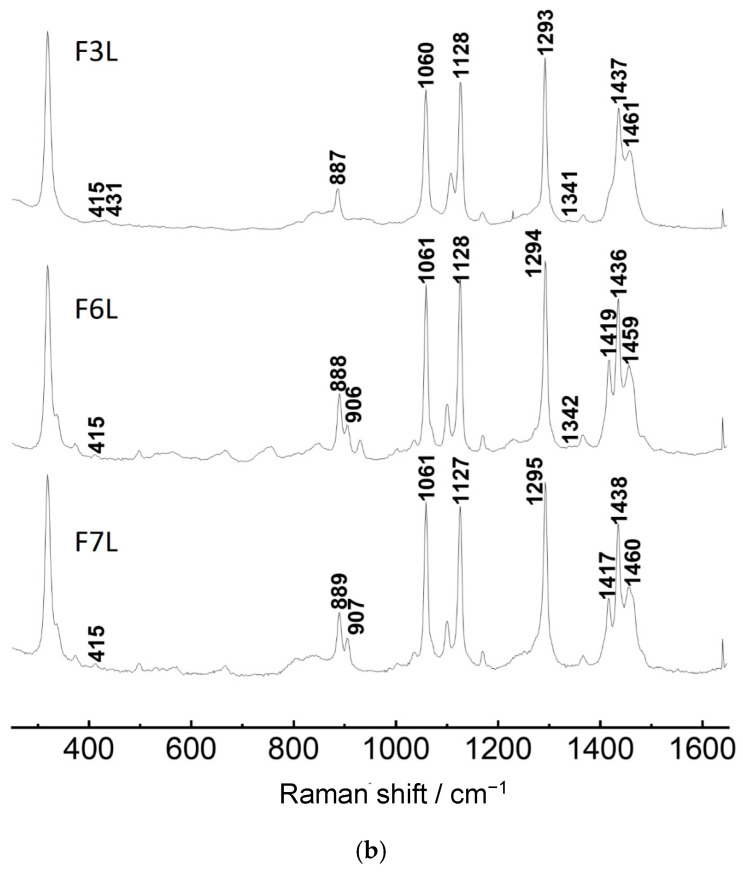
Raman spectra of (**a**) API or excipients and (**b**) SLM formulations over the range 200 cm^−1^–1600 cm^−1^.

The spectra on Figure 5a are the reference spectra from the pure substances. The recorded spectra showed a clear presence of the peaks characteristic for the lipids. The strongest peaks were situated at 1060–1062 cm^−1^ (C–C asymmetric stretching), 1128–1130 cm^−1^ (C–C symmetric stretching) and 1294–1295 cm^−1^ (CH_2_ twisting) [30,31]. At the same time, among the characteristic bands distinguished for CsA and SPIR (417, 1340, 635, 1620 and 952 cm^−1^), the diagnostic bands were selected in such ranges in which they did not coincide with those from lipids or other excipients. Unfortunately, the signals from CsA and SPIR on the spectra of SLM (Figure 5b) did not have enough intensity compared to the lipid bands, even if the concentration of CsA was 5% (*w/w*).

**Figure 6:**
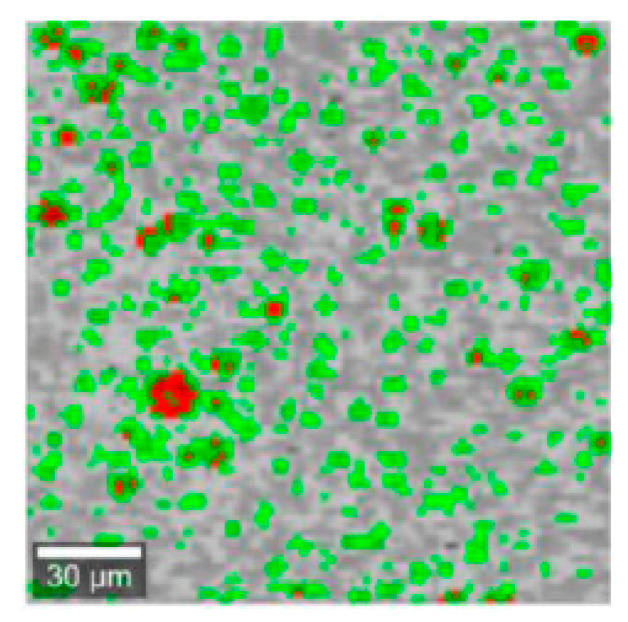
Raman maps (20× resolution) of F3L prepared “de novo” microparticles formulations. Red and green correspond to the API and lipids, respectively.

Preliminary Raman mapping was performed with a magnification of 20×, generating a spot size of approximately 3 μm. The red spots (Figure 6) observed on the maps of selected formulations (F2–F3 and F7–F8) correspond to the characteristic CsA peaks. Figure 5b depicts the example spectra from 1600 to 200 cm^−1^ extrapolated from the Raman maps. Some CsA characteristic peaks can be identified at 415 cm^−1^ and 1340 cm^−1^, but they are almost undetectable, because their intensity is very small compared to the lipid-derived bands (1061, 1126 and 1293 cm^−1^). It should also be remembered that PVP was included in the composition (Table 1) of the dried formulations (added before drying), which further increased the complexity of the tested FS formulations.

**Figure 7:**
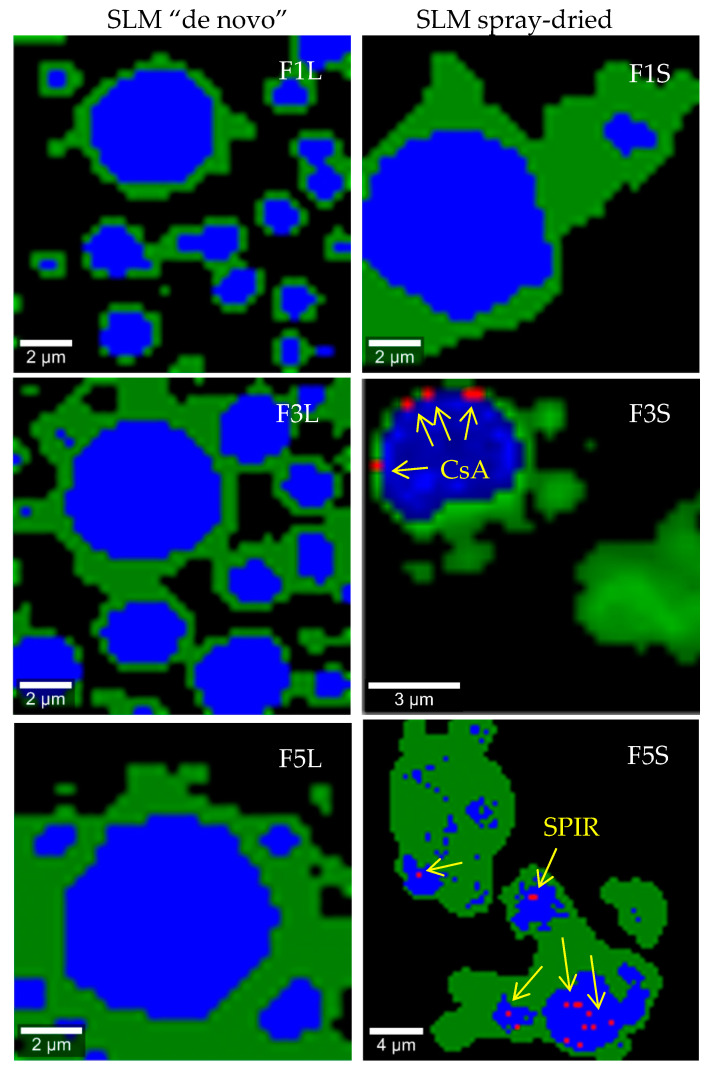

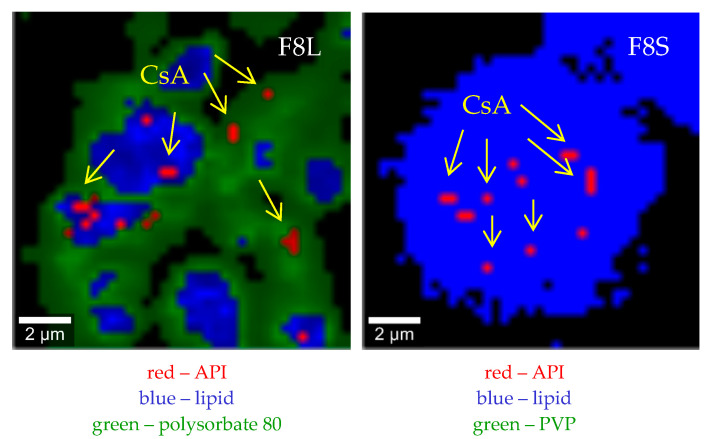
Raman maps (100× resolution) of SLM placebo and with the API formulations prepared “*d*e novo” (FL) and after spray drying (FS) with Compritol (F1, F3 and F5) or stearic acid (F8).

To make the surface of single SLM more visible and to improve the detection of the API, the magnification was changed to 100×, with a spot size of approximately 0.3 μm. When 100× magnification was applied, intense signals from the lipids (blue color on the maps) were obtained, and they clearly assumed spherical shapes of the lipid particles. The green color on the maps originate from polysorbate in liquid or PVP in spray-dried formulations (Figure 7). When FL and FS formulations, the placebo and the API-loaded were compared, no substantial differences were observed. Figure 7 shows the distribution maps of the API in the selected SLM formulations at 100× magnification. The red spots observed on the Raman maps (Figure 7) of the liquid or spray-dried formulations (F1–F10) correspond to the presence of a characteristic CsA or SPIR peak at 417 and 1620 cm^−1^, respectively.

Figure 7 reveals that, in some drug-loaded SLM formulations (i.e., F3L and F5L), the spectral features of CsA or SPIR (Figure 5a) were not recognized. Since the distribution and release studies did not show clear differences after drying in these formulations, this may indicate that simple univariate imaging does not provide reliable maps of CsA or SPIR on the SLM surface and may be misleading. At this stage of the research, this indicates the need to verify the conclusions obtained on the basis of the Raman maps analysis. Better results would be expected at a higher API concentration or when testing another API with characteristic spectral properties.

### 3.5. Proton Nuclear Magnetic Resonance (^1^H NMR) Measurements

NMR spectroscopy is one of the methods to study molecular structures in detail. NMR is an analytical technique that uses radiofrequencies to detect atomic nuclei (^1^H) in molecules. ^1^H NMR studies were performed to further select the methods that may be useful in assessing the interactions of the components of SLM and provide new information on the changes occurring under the influence of spray drying.

The studies were based on the assumption that the lipid-API interactions and/or expulsion of the API will be reflected by the appearance of signals characteristic for API or by the change of the widths or amplitudes of the signals generated by the tested SLM. As already described in the case of other solid lipid particles, NMR signals related to triglycerides are difficult to observe due to the very short relaxation times [55,56].

As already mentioned, a different localization of the API in SLM should be taken into consideration. Moreover, the relocation of the API molecules may occur, especially during storage or reprocessing (spray drying). ^1^H NMR is an appropriate tool to distinguish between mobile liquid and immobilized solid states, because the line’s width of the NMR signal is related to the phenomenon of relaxation, which depends on the degree of mobility of the molecules [29]. Therefore, it was expected that this method would allow the possible detection of the mobility and rearrangement of the API molecules excluded from the matrix, if such an event appeared.

**Figure 8:**
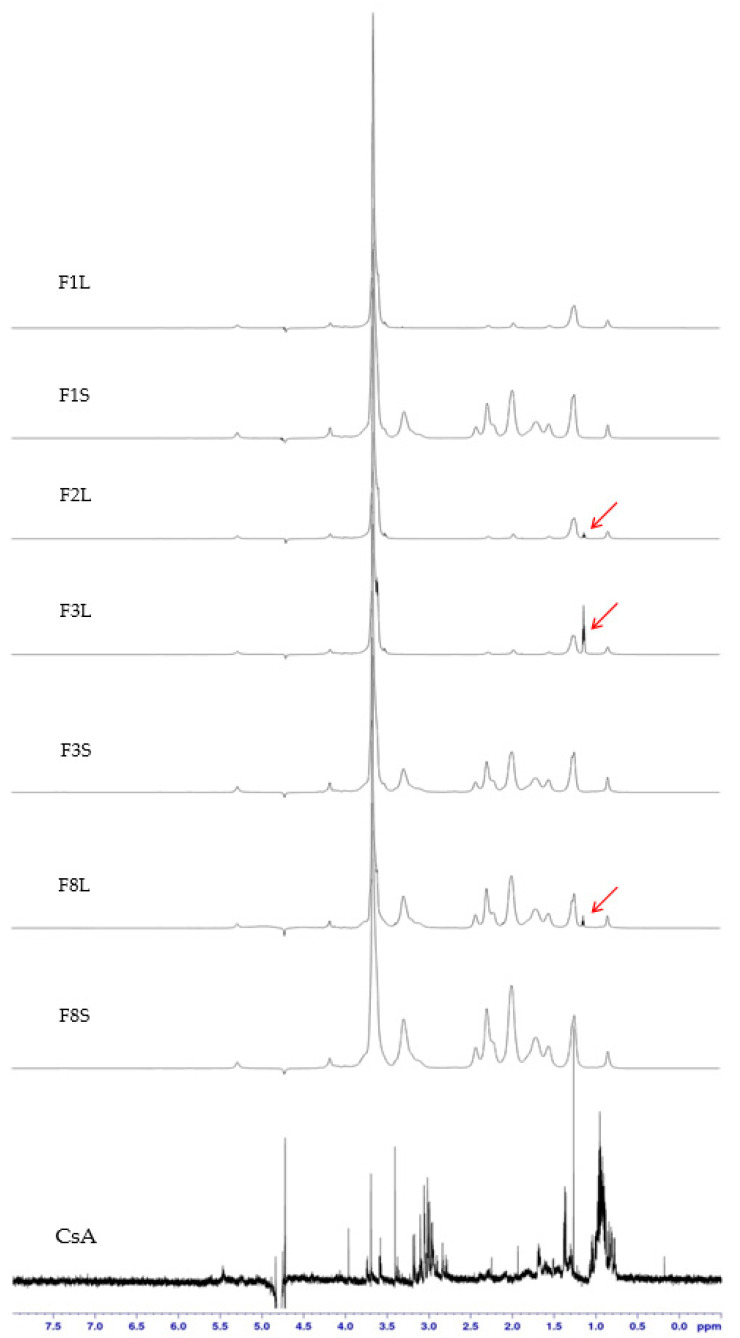
High-resolution proton nuclear magnetic resonance (^1^H NMR) spectra of the SLM placebo and with CsA prepared “de novo” (FL) and after spray drying (FS) with Compritol (F1–F3) or stearic acid (F8).

Examples of the spectra of the API, placebo SLM and SLM with the API are shown in Figure 8. The comparison of the fingerprint regions (2.8–3.3 ppm) obtained from the CsA and CsA-SLM reveals the lack of characteristic signals from CsA in both liquid and dry formulations. The effect of CsA incorporation becomes noticeable only in the 1.0–1.2 ppm region, when NMR spectra were analyzed over the entire range (F2L, F3L and F8L in Figure 8). An additional band with the intensity increasing with the increased CsA concentration was identified irrespective of the type of lipid. This line is invisible, however, in the spectra of spray-dried formulations. At the same time, in the mentioned spectra of the dried formulations, there are numerous additional bands in the range of 1.0–3.5 ppm (F1S, F3S and F8S in Figure 8), which result from the presence of PVP added before spray drying. To confirm this, PVP was also added to the non-spray-dried SLM (F6L and F8L formulations), and the same bands in their NMR spectra were detected. Similar dominating signals from other additives, like polyvinyl alcohol or poloxamer, were observed when SLN formulations were analyzed [29,55].

No change in the NMR spectrum of SLM was observed due to the presence of SPIR. Moreover, the spectra of SPIR-SLM before or after the spray drying did not differ (besides the above-mentioned bands from PVP). This fact confirms that the drying process does not affect the formulation properties. The lack of SPIR signals in the diagnostic range of the NMR spectra (fingerprint region) indicates its binding in SLM lipid particles, as in the case of CsA.

If only mobile particles of the API can appear as bands in the NMR spectrum, one can assume that the band associated with the appearance of CsA in the formulation results from the presence of a small fraction of the API (about 1%) in the aqueous phase of the SLM dispersion (see Table 2). The disappearance of this band in dried microspheres in this situation is associated with the absence of CsA in the solution (in dissolved form). When considering the results of other studies, the persistent binding of the API to lipids in SLM, resulting in their restricted mobility, is the main cause responsible for the observed results [55,56].

SPIR, unlike CsA, is poorly soluble in oil and incorporates in the lipid matrix of SLM not so effectively (see Section 3.1). Therefore, it is surprising that there are no changes in the NMR spectra of the SPIR formulation, even though its amount in the aqueous phase is several times greater than CsA.

One can conclude that, when applying ^1^H NMR spectroscopy, there is no significant effect of the spray-drying process on the structure of SLM. Unfortunately, it is difficult to identify signals of the API if it is incorporated in the solid lipid matrix.

### 3.6. Atomic Force Microscopy (AFM) Measurements

In the vast majority of publications, AFM was used only to image the surface topography of SLN and to show the shapes and sizes of these particles [29], although, using this technique, one can also measure the viscoelastic properties of SLM surfaces based on the interactions of the particle surface with the probe. Introducing the AFM into our spectrum of the physical analysis of the SLM, we aimed at an insight into the interaction of the lipid matrix with the API and its impact on the localization of the API based on a change in these properties.

**Figure 9:**
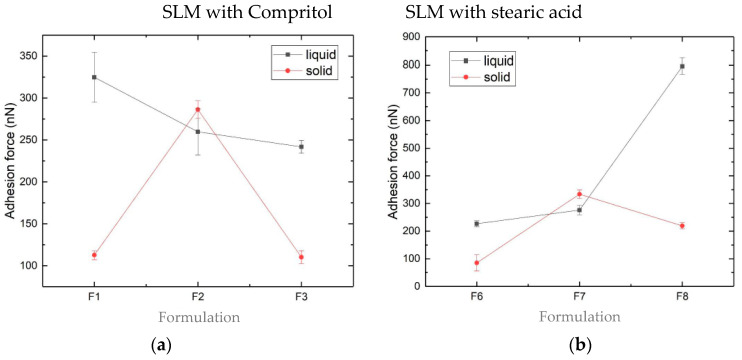

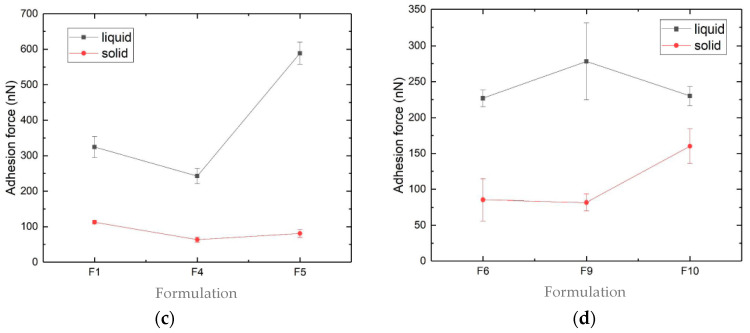
Average adhesion forces measured by the atomic force microscopy (AFM) of the SLM placebo and with the API formulations prepared “de novo” (■ liquid) and after spray drying (● solid) with Compritol: (**a**) and (**c**) or with stearic acid: (**b**) and (**d**).

In the performed studies, all the measurements were repeated eight times. Each cycle was manifested by a well-defined discontinuity in the force-distance approach curves, which was subsequently analyzed to determine the magnitude of the adhesion force. Figure 9 presents the average values of the adhesion force for all the tested formulations.

At the current stage of research, a large range of the measured forces (from 63 nN even to 795 nN) was observed. In liquid formulations with Compritol (Figure 9a,c), the increased concentrations of CsA and SPIR resulted, respectively, in decreased and increased adhesion strengths. In the formulations with stearic acid, the reverse tendency was seen (Figure 9b,d). As a rule, smaller adhesion strengths were measured in the spray-dried formulations. Visible differences in the values of the adhesion forces (Figure 9) reflect differences in the surface properties of the tested SLM, which may be due to the presence or absence of the API.

The results obtained indicate significant potential in the application of the AFM method to characterize the surfaces of lipid particles. Unfortunately, at the current stage of research, because of too many formulation variables and due to an insufficient number of experiments, conclusions cannot be clearly defined yet, although some trends are already visible. In addition, the results obtained largely depend on the selected probe. Therefore, maintaining the observed trends using a different probe would also require confirmation.

## 4. Conclusions

Selected analytical methods were introduced to detect possible lipid-API interactions and changes in the physicochemical properties of the tested SLM formulations after the spray-drying process. It has been shown that the spray drying did not affect the properties of the majority of the tested formulations. However, that depended mainly on the type of lipid and the API. In the distribution and release studies, some differences were apparent, mainly in SLM with Compritol and with lower API concentrations. As a rule, the distribution and drug release data were compatible, though some discrepancies were observed in SLM sensitive to drying.

DSC is one of the primary tools used for the characterization of lipid polymorphism and drug-lipid interactions. This method allowed to detect some changes in SLM with stearic acid, which was justified by a greater sensitivity of this lipid to temperature during drying.

Raman spectroscopy is a useful technique, as it involves no sample preparation and, most importantly, allows measurements in the presence of water. However, at the current stage of our research, it has not brought the expected results, and the attempt to confirm with this technique the localization of the API on the surface of the SLM was unsuccessful. Although both CsA and SPIR were identified on the SLM surface, the dominant components on the Raman maps were lipids and polysorbate or PVP. Discrimination on the spectra of the bands derived from the API and lipids (or other excipients) was impossible, mainly due to the spectral properties of the tested API and their low concentrations in the formulations.

Some differences in the ^1^H NMR spectra of the placebo and CsA-loaded SLM were only visible in the liquid formulations (FL) but not in the typical diagnostic range, and no difference was found in SPIR-SLM. The characteristic signal in the FL spectra disappeared in the FS spectra, which allowed to differentiate the spectra of the formulations before and after the drying process and indicated the effects of spray drying on the tested SLM formulations. Although the ^1^H NMR has the potential to detect changes in the physicochemical properties of the SLM caused by spray drying, it highly depends on the properties of the API.

The nanomechanical properties of the SLM surface investigated with AFM provide the possibility to differentiate between the SLM with and without the API on the lipid surfaces, as well as to observe changes occurring under the influence of spray drying. The advantage of the AFM method is that the sample preparation is simple and not time-consuming. At the same time, this method is more demanding—for example, in terms of selecting the appropriate probe or localization, in which the probe-sample interaction is measured. Our results, although not being conclusive, justify further investigations of the SLM with the AFM technique not only for obtaining topographical images but, also, to analyze the distribution of the API on the surfaces of lipid microparticles.

In conclusion, all of the used instrumental analytical methods are complementary and provide different information, more or less characterizing the examined SLM as multicompartment system. Particularly, the continuation of AFM research and further attempts to use Raman spectroscopy are justified. At this stage, however, DSC, AFM and Raman spectroscopy were recognized as the most valuable, and due to the above-mentioned limitations, NMR was classified as a second-choice technique. Nevertheless, for each of the employed analytical techniques, a very careful selection of the measurement parameters and proper analytical procedure are crucial to obtain reliable and reproducible test results, especially if material such as the SLM in dispersion or a powder form is tested. At the same time, the range of analytical techniques employed in our study does not exhaust all methods that could be used to assess the physicochemical properties of the SLM and occurring interactions. Therefore, in addition to the aforesaid continuation of AFM and Raman analyses, other techniques, such as X-ray photoelectron spectroscopy, will also be considered.

## Figures and Tables

**Table 1:** The composition (*w/w* %) of the investigated formulations before (FL—liquid formulations) and after (FS—spray-dried formulations) spray drying. CsA: cyclosporine A, SPIR: spironolactone and PVP: polyvinylpyrrolidone.

Formulation		The Composition of Formulations (FL and FS)													
		CsA		SPIR		Compritol		Stearic Acid		Tween 80		Water		PVP	
FL	FS	FL	FS	FL	FS	FL	FS	FL	FS	FL	FS	FL	FS	FL	FS
F1L	F1S	-	-	-	-	10.0	50.0	-	-	5.0	25.0	85.0	-	-	25.0
F2L	F2S	0.1	0.5	-	-	10.0	49.7	-	-	5.0	24.9	84.9	-	-	24.9
F3L	F3S	1.0	4.8	-	-	10.0	47.6	-	-	5.0	23.8	84.0	-	-	23.8
F4L	F4S	-	-	0.1	0.5	10.0	49.7	-	-	5.0	24.9	84.9	-	-	24.9
F5L	F5S	-	-	0.5	2.4	10.0	48.8	-	-	5.0	24.4	84.5	-	-	24.4
F6L	F6S	-	-	-	-	-	-	10.0	55.5	3.0	16.7	87.0	-	-	27.8
F7L	F7S	0.1	0.6	-	-	-	-	10.0	55.2	3.0	16.6	86.9	-	-	27.6
F8L	F8S	1.0	5.3	-	-	-	-	10.0	52.6	3.0	15.8	86.0	-	-	26.3
F9L	F9S	-	-	0.1	0.6	-	-	10.0	55.2	3.0	16.6	86.9	-	-	27.6
F10L	F10S	-	-	0.5	2.7	-	-	10.0	54.1	3.0	16.2	86.5	-	-	27.0

**Table 2:** Characteristics of the investigated solid lipid microparticle (SLM) dispersions prepared “de novo” (FL—liquid formulations) and reconstituted (FS—spray-dried formulations). API: active pharmaceutical ingredient, d _0.5_ and d _0.9_: the particle size at which the cumulative particle size distribution curve reaches 50% and 90% of the volume, respectively.

Formulation		Particle Size (µm)		pH	API Distribution (%)						API Release (%) ^2^			
		d _0.5_	d _0.9_		Aqueous Phase		Interphase		Lipid Matrix		0.5 h ^2^		24 h ^2^	
FL	FS	FL	FL	FL	FL	FS	FL	FS	FL	FS	FL	FS	FL	FS
F1L	F1S	1.6 ± 0.01	5.7 ± 0.22	5.44 ± 0.14	-	-	-	-	-	-	-	-	-	-
F2L	F2S	1.6 ± 0.01	6.1 ± 0.40	5.71 ± 0.08	0.1 ± 0.04	0.1 ± 0.15	52.1 ± 7.8	69.3 ± 8.2	47.8 ± 7.9	30.6 ± 8.3	21 ± 0.8	65 ± 4.9	62 ± 0.6	80 ± 3.7
F3L	F3S	1.8 ± 0.09	6.4 ± 0.58	5.82 ± 0.15	0.1 ± 0.01	0.4 ± 0.14	70.9 ± 10.4	72.6 ± 6.7	29.0 ± 10.4	27.0 ± 6.8	50 ± 3.1	66 ± 3.6	67 ± 1.8	81 ± 2.4
F4L	F4S	2.3 ± 0.66	6.0 ± 0.74	5.29 ± 0.20	2.5 ± 3.43	4.2 ± 2.77	76.6 ± 1.8	67.5 ± 4.3	20.9 ± 3.1	28.3 ± 4.3	13 ± 6.2	81 ± 5.3	100 ± 2.6	89 ± 5.7
F5L	F5S	4.4 ± 1.67	10.5 ± 4.88	5.48 ± 0.09	0.3 ± 0.44 ^1^	0.4 ± 0.56 ^1^	24.9 ± 12.6 ^1^	50.1 ± 5.0 ^1^	74.8 ± 11.9 ^1^	49.5 ± 5.4 ^1^	25 ^1^ –	54 ^1^ –	89 ^1^ –	82 ^1^ –
F6L	F6S	2.2 ± 0.36	5.7 ± 0.30	5.30 ± 0.10	-	-	-	-	-	-	-	-	-	-
F7L	F7S	2.3 ± 0.05	5.8 ± 0.20	5.63 ± 0.11	1.3 ± 1.79	2.0 ± 2.79	76.3 ± 8.6	73.8 ± 6.2	22.4 ± 9.3	24.2 ± 5.3	53 ± 2.9	59 ± 1.5	63 ± 4.2	63 ± 5.7
F8L	F8S	6.4 ± 0.17	14.6 ± 0.51	5.93 ± 0.23	0.6 ± 0.77	0.1 ± 0.08	73.7 ± 3.3	73.5 ± 4.5	25.7 ± 3.5	26.4 ± 4.5	73 ± 2.8	88 ± 3.0	94 ± 1.9	90 ± 4.3
F9L	F9S	2.1 ± 0.54	7.2 ± 1.85	5.21 ± 0.11	3.6 ± 1.05	4.1 ± 1.42	39.6 ± 1.1	46.8 ± 2.1	56.8 ± 0.9	49.1 ± 2.2	53 ± 6.8	50 ± 3.9	50 ± 12.4	94 ± 0.2
F10L	F10S	6.4 ± 1.01	14.6 ± 1.43	5.24 ± 0.07	0.6 ± 0.03 ^1^	0.5 ± 0.06 ^1^	43.7 ± 33.7 ^1^	70.4 ± 3.2 ^1^	55.7 ± 32.8 ^1^	29.1 ± 3.1 ^1^	8 ^1^ –	73 ^1^ –	21 ^1^ –	73 ^1^ –

^1^ Incomplete incorporation of spironolactone (SPIR) during preparation (active substance crystals in dispersions were observed). Data are presented as mean ± SD (*n* = 3 and ^2^ *n* = 2).
